# Comparative analysis of carboplatin and paclitaxel combination chemotherapy schedules in previously untreated patients with advanced non-small cell lung cancer

**DOI:** 10.3892/ol.2013.1134

**Published:** 2013-01-15

**Authors:** TOSHIKI SHIMIZU, TAKASHI YOKOI, TAKESHI TAMAKI, KAYOKO KIBATA, NORIKO INAGAKI, SHOSAKU NOMURA

**Affiliations:** First Department of Internal Medicine, Kansai Medical University, Moriguchi, Osaka 570-8506, Japan

**Keywords:** non-small cell lung cancer, weekly, carboplatin, paclitaxel, neutropenia

## Abstract

The combination of carboplatin and paclitaxel is one of the most commonly used regimens for the treatment of non-small cell lung cancer (NSCLC). We aimed to compare the standard tri-weekly and weekly schedules of this treatment, while considering treatment-related hematological toxicities. We retrospectively analyzed the weekly [paclitaxel, 70 mg/m^2^/week on days 1, 8 and 15, and carboplatin, area under the curve (AUC)=6, every 4 weeks] and standard tri-weekly (paclitaxel, 200 mg/m^2^, and carboplatin, AUC=6, on day 1 every 3 weeks] schedules in patients with previously untreated advanced NSCLC. A total of 167 patients were enrolled in this study. The median age of the patients was 65 years (range, 31–79 years). The weekly and standard arms included 73 and 94 patients, respectively. The incidence of grade 3 or 4 neutropenia and neuropathy was significantly decreased in the weekly arm compared with the standard arm (37.0 vs. 70.2%). The median survival and progression-free survival times were 11.8 and 4.2 months, respectively, in the weekly arm and 11.6 and 3.1 months, respectively, in the standard arm. The results of the multivariate analysis indicated that the weekly schedule [hazard ratio (HR)=0.634, P=0.0262] and grade 3 or 4 neutropenia (HR=0.372, P=0.0007) were independent favorable prognostic factors for overall survival time. In conclusion, the weekly schedule of carboplatin and paclitaxel was less toxic than and potentially superior to the standard tri-weekly schedule. However, further optimization of the dose and schedule is warranted.

## Introduction

Lung cancer is the leading cause of cancer-related mortality worldwide. Platinum-based chemotherapy is recommended as a front-line chemotherapy for advanced non-small cell lung cancer (NSCLC) (1). The results of the Four Arms Comparative Study, which analyzed 4 platinum-doublet regimens as front-line therapies for advanced NSCLC, demonstrated that the 4 regimens had similar efficacy (2,3). Thus, carboplatin and paclitaxel combination therapy has been recognized as a reference regimen based on its feasibility. In the standard schedule, 200 mg/m^2^ paclitaxel and carboplatin [area under the curve (AUC)=6] are administered on day 1 every 3 weeks. However, several weekly schedules have been investigated to reduce hematological toxicities (4–6). Hirabayashi *et al* also reported a weekly schedule based on the metronomic theory (7). In this weekly schedule, 70 mg/m^2^ paclitaxel on days 1, 8 and 15, and AUC=6 of carboplatin on day 1 were administered every 4 weeks. All the studies, including the present study, demonstrated that a weekly schedule reduced neutropenia and displayed a comparable survival benefit compared to the standard schedule.

The survival benefit of treatment-related neutropenia has been discussed in previous studies (8–11). Consequently, a treatment schedule correlated with a reduced incidence of neutropenia ought to be an unfavorable predictive factor for overall survival (OS). However, a weekly schedule associated with reduced neutropenia demonstrated a comparable survival benefit to the standard schedule. Notably, there has been no integrated analysis comparing the 2 schedules and concurrently considering the worst grade of treatment-related hematological toxicities. Therefore, we performed a retrospective analysis in unselected patients who received standard tri-weekly or weekly carboplatin in combination with paclitaxel to obtain complimentary information regarding whether the treatment schedule and treatment-related hematological toxicities were correlated with survival outcome.

## Patients and methods

### Data collection

The medical records of all patients with NSCLC who were treated between January 1999 and December 2010 at Kansai Medical University Takii Hospital (Moriguchi, Japan) were retrospectively reviewed; institutional review board approval was obtained for this study. The patients were included in this study if they had advanced NSCLC (stage IIIB or IV) that was treated with front-line combination chemotherapy including carboplatin and paclitaxel. The patients were assigned to 1 of 2 groups; the weekly arm group (weekly schedule) and the standard arm group (standard tri-weekly schedule). The clinical stage was assigned on the basis of the Sixth Edition of the TNM Classification for Lung Cancer (12,13). Data including gender, age, smoking history, clinical stage, histological cancer type, history of administration of an epidermal growth factor receptor tyrosine kinase inhibitor (EGFR-TKI), Eastern Cooperative Oncology Group (ECOG) performance status (PS), progression-free survival (PFS) and OS were obtained retrospectively from the patient medical records. There was no distinction between gefitinib and erlotinib, and both agents were considered as EGFR-TKIs. Patients who underwent previous palliative radiation treatment, including whole-brain irradiation without curative intention, were included. The crossover cases in the consecutive treatment courses between the weekly and standard arms were excluded, as well as patients with large cell neuroendocine carcinoma. All patients provided informed consent prior to receiving front-line chemotherapy. The study was performed according to the Declaration of Helsinki and was approved by the Institutional Ethics Review Board (the Clinical Research Board of Kansai Medical University Takii Hospital; ID No, 23-6).

### Statistical analysis

Differences between the groups were compared using the χ^2^ or Fisher’s exact test. OS was defined as the time from the start of front-line systemic chemotherapy to the time of death from any cause or the date the patient was last known to be alive. PFS was defined as the time between the start of treatment and disease progression, death or the last known follow-up. The treatment-related adverse effects were evaluated using the Common Terminology Criteria for Adverse Events version 4.0 (14). Objective tumor responses to chemotherapy were evaluated using the Response Evaluation Criteria in Solid Tumors version 1.0 (15). The objective response rate (ORR) was defined as the number of patients displaying a complete response (CR) or a partial response (PR), with respect to the total number of patients evaluated. The disease control rate (DCR) was defined as the number of patients displaying a CR, a PR or stable disease (SD), with respect to the total number of patients evaluated. The minimum time interval between the 2 measurements required for the determination of SD was 6 weeks. The 95% confidence intervals (95% CIs) for the ORR and DCR were calculated using a binomial distribution. The univariate and multivariate analyses of PFS and OS were performed with the Kaplan-Meier product-limit method using the log-rank test and the Cox proportional hazards model, respectively. The 95% CI for the survival rate was calculated using Greenwood’s method, and that of the median survival time (MST) was caluclated by the Brookmeyer and Crowley method. All statistical analyses were conducted using JMP (version 9.0.2) software for Windows (SAS Institute Inc, Cary, NC). All statistical tests were two-sided, and P<0.05 was considered to indicate a statistically significant difference.

### Treatment plan

In the weekly arm (weekly schedule), 70 mg/m^2^ paclitaxel was administered on days 1, 8 and 15 together with carboplatin (AUC=6) on day 1 of each 4-week cycle. In the standard arm (standard tri-weekly schedule), 200 mg/m^2^ paclitaxel was administered with carboplatin (AUC=6) on day 1 of each 3-week cycle. Thirty minutes prior to paclitaxel administration, the patients were treated with the following premedications: dexamethasone (20 mg), diphenhydramine (50 mg and a histamine receptor 2 (H2) blocker. The patients in the weekly arm were permittted to have their premedications altered to dexamethasone (8 mg), diphenhydramine (50 mg) and an H2 blocker. The glomerular filtration rate was substituted by the calculated value using the Cockroft equation. The treatment was continued up to a maximum of 6 cycles or until disease progression in both arms.

## Results

### Patient characteristics

A total of 402 patients with NSCLC were treated in our hospital between January 1999 and December 2010. Of the 218 patients with advanced-stage disease, 167 met the eligibility criteria. The characteristics of these 167 patients are summarized in Table I. All patients were Asian (Japanese, Korean or Chinese), the median patient age was 65 years (range, 31–79 years) and patients comprised 42 females and 125 males. The numbers of patients with adenocarcinoma, squamous cell carcinoma, large cell carcinoma and other types of carcinoma were 118, 44, 4 and 1, respectively. The weekly and standard arm regimens were used as front-line chemotherapies in 73 and 94 patients, respectively. In 108 patients, ≥1 regimen of chemotherapy, including EGFR-TKI, was administered following the front-line chemotherapy. A history of EGFR-TKI treatment was reported in 66 patients, whereas the remaining 101 patients had not received EGFR-TKI treatment. There were no significant differences in age, gender, PS, clinical stage and smoking history between the 2 groups.

### Treatment-related adverse effects

The treatment-related grade 2 or worse adverse effects observed in this study are summarized in Table II. Grade 3 or 4 neutropenia was observed in 37.0 and 70.2% of the patients in the weekly and standard arms, respectively, indicating that severe (grade 3 or 4) neutropenia was significantly more frequent in the standard arm (P<0.0001). Grade 3 or 4 thrombocytopenia was observed in 11.0 and 12.8% of the patients in the weekly and standard arms, respectively (P=0.8127). Grade 3 or 4 peripheral neuropathy was observed in 0% and 8.5% of the patients in the weekly and standard arms, respectively (P=0.0188). Grade 4 neuropathy was not observed in the weekly arm, although it was observed in 3.2% of patients in the standard arm. The other hematological or non-hematological adverse effects observed were found to be moderate and manageable in both groups.

### Tumor response

The tumor responses are listed in Table I. The ORRs of the weekly and standard arms were 37.0% (95% CI, 26.0–49.1) and 31.9% (95% CI, 22.7–42.3), respectively. The DCRs of the weekly and standard arms were 74.0% (95% CI, 62.4–83.6) and 74.5% (95% CI, 64.4–82.9), respectively. There were no significant differences in ORR and DCR between the 2 groups.

### Survival data

**Figure 1:**
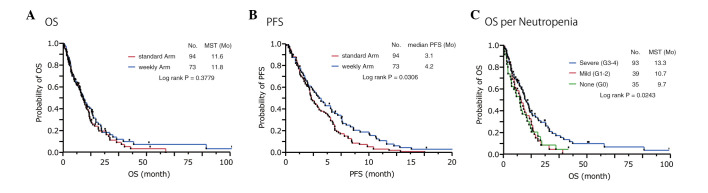
Kaplan-Meier curves of patients treated with carboplatin in combination with paclitaxel. (A) The Kaplan-Meier curves for overall survival (OS). The median survival times (MSTs) for the weekly and standard arms were 11.8 and 11.6 months, respectively (log-rank; P=0.3779). (B) The Kaplan-Meier curves for progression-free survival (PFS). The median PFS times for the weekly and standard arms were 4.2 and 3.1 months, respectively (log-rank; P=0.0306). (C) The Kaplan-Meier curves for OS according to the worst grade of treatment-related neutropenia. The MSTs for severe (grade 3 or 4), mild (grade 1 or 2) and absent (grade 0) neutropenia were 13.3, 10.7 and 9.7 months, respectively (log-rank; P=0.0243).

We conducted a series of survival analyses on September 1, 2012. At that time, 136 patients had died, 28 patients had been lost to follow-up and 3 patients were alive. Consequently, the censoring rate was estimated at 18.6%. The MSTs were 11.8 months (95% CI, 8.4–16.8) and 11.6 months (95% CI, 9.5–14.6) for the patients in the weekly and standard arms, respectively (Fig. 1A). The 1-year survival rates were 49.2% (95% CI, 37.1–61.3) and 48.8% (95% CI, 38.3–59.2) for the patients in the weekly and standard arms, respectively. The median PFS times were 4.2 months (95% CI, 2.7–5.5) and 3.1 months (95% CI, 2.8–4.0) for the patients in the weekly and standard arms, respectively (Fig. 1B). In the univariate analyses, the PFS was significantly longer in the weekly arm group (P=0.0306). The hazard ratio (HR) for the PFS of patients in the weekly arm versus the standard arm was 0.576 (P=0.0024).

### Univariate analyses for OS

In the univariate analyses, the OS was significantly longer in the patients treated with EGFRTKIs (P=0.0007), females (P=0.0051), individuals who had never smoked (P=0.0043), those with PS 0/1/2 (P=0.0062), those with nonsquamous cell carcinoma (P=0.0280) and those with stage IIIB disease (P=0.0248), compared with the respective counterparts. However, the front-line chemotherapy schedules (P=0.3779) and patient age (P=0.6135) were not statistically significant prognostic factors for OS (Table III). We also analyzed the contribution of the worst grade of treatment-related hematological toxicity to OS. The correlation between OS and thrombocytopenia was not statistically significant (P= 0.3718). The OS was significantly shorter in patients with severe anemia (grade 3 or 4; P=0.0002). On the contrary, the results of the alternative analyses clearly indicated that OS was significantly longer in patients with severe (grade 3 or 4) neutropenia (P=0.0243; Fig. 1C).

**Figure 2:**
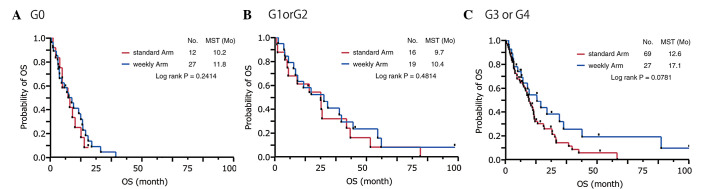
Kaplan-Meier curves of the patients according to the worst grade of treatment-related neutropenia. (A) The Kaplan-Meier curves for overall survival (OS) for the subgroup with grade 0 neutropenia. The median survival times (MSTs) for the weekly and standard arms were 11.8 and 10.2 months, respectively (log-rank; P=0.2414). (B) The Kaplan-Meier curves for OS for the subgroup with grade 1 or 2 neutropenia. The MSTs for the weekly and standard arms were 10.4 and 9.7 months, respectively (log-rank; P=0.4814). (C) The Kaplan-Meier curves for OS for the subgroup with grade 3 or 4 neutropenia. The MSTs for the weekly and standard arms were 17.1 and 12.6 months, respectively (log-rank; P=0.0781).

We had demonstrated that severe neutropenia more frequently occurred with the standard schedule (70.2 vs. 37.0%), which indicated a significant survival impact, than in the weekly schedule; however, the survival curves of the 2 schedules almost overlapped (Fig. 1A). Subsequently, we conducted an alternative subanalysis for OS according to the worst grade of neutropenia (Fig. 2). In the subpopulations of mild (grade 1 or 2) and no (grade 0) neutropenia, comparable survival was demonstrated for the 2 schedules (Fig. 2A and B). However, in the subpopulation of severe (grade 3 or 4) neutropenia, the patients in the weekly schedule arm tended to have better survival that those in the standard schedule arm (P=0.0781; Fig. 2C). To evaluate the independent survival impact of the covariates, we subsequently conducted a multivariate analysis that included the severity of hematological toxicities as covariates.

### Multivariate analysis for OS

Unexpectedly, the multivariate analysis revealed that the weekly schedule was an independent favorable prognostic factor for OS (HR=0.634; P=0.0262), whereas the results of the univariate analysis failed to indicate a significant difference. In addition, PS 0/1/2 (P=0.0002), stage IIIB disease (P=0.0011), a history of EGFR-TKI treatment (P=0.0007), female gender (P=0.0320), grade 3 or 4 neutropenia (P=0.0002) and grade 0 anemia (P<0.0001) were also independent favorable prognostic factors (Table IV). However, no significant difference in OS was observed between patients with grade 1 or 2 neutropenia and those with grade 0 neutropenia (P=0.5392).

## Discussion

The weekly schedule of paclitaxel and carboplatin combination treatment was investigated with 2 aims: one was to reduce the incidence of treatment-related toxicities, and the other was to assess the alternative antitumor effect based on the metronomic theory (16). To date, several weekly treatment schedules have been investigated (4–6). We applied the weekly schedule scheme (weekly arm) demonstrated by Hirabayashi *et al*(7). The efficacy and feasibility of this regimen were confirmed in an alternative phase II study described by Komuta *et al*(17). A previous randomized study described by Sakakibara *et al* compared these 2 schedule plans in an elderly patient (≥70 years of age) setting and observed that in addition to its comparable survival benefit, the weekly schedule was less toxic than the standard schedule (18).

However, the close correlation between chemotherapy-related hematological toxicity and survival benefit has been discussed extensively for various malignant tumors (8–11). Kishida *et al* demonstrated that treatment-related neutropenia was a favorable predictive factor for the OS of patients with advanced NSCLC (JMTOG LC00-03) (9). Two additional studies have supported these findings (10,11). We also demonstrated that grade 3 or 4 neutropenia was an independent favorable prognostic factor for OS in this study. However, the results of our univariate analyses clearly demonstrated that the OS times were similar between the 2 treatment schedules, whereas the incidence of grade 3 or 4 neutropenia was significantly lower in the weekly schedule arm than in the standard schedule arm. We hypothesized that an alternative survival benefit arising from the weekly schedule may overcome the disadvantage of the decreased incidence of neutropenia in that group. One possible explanation for this finding is that the metronomic activity of the weekly schedule may result in an alternative antitumor effect, leading to an additional survival benefit. Low-dose metronomic (LDM) chemotherapy consists of the administration of a relatively low dose of a cytotoxic drug without a long interval (19,20). A number of experimental studies have demonstrated that LDM chemotherapy exerts an alternative antitumor effect different from the direct cytotoxic effect of the drug (16,21). Notably, the result of our multivariate analysis revealed that the weekly schedule was an independent favorable prognostic factor for OS. Based on our rationale, optimization of the dose and schedule ought to achieve a superior survival benefit for the weekly schedule. Katsumata *et al* clearly demonstrated that a myelosuppressive ‘dose-dense’ weekly schedule has a superior survival benefit for ovarian cancer compared with the standard tri-weekly schedule (22). These findings strongly support our hypothesis. Therefore, the next question is whether escalation of the relative dose density leads to superior survival benefits. This will not be consistently true because the metronomic power is not augmented in a dose-dependent manner. The maximum metronomic power of a drug is defined by the optimal biological dose (OBD) rather than the maximum tolerated dose (23). There is no reliable surrogate marker to determine OBD.

Despite the retrospective nature and small scale of the present study, our results clearly demonstrated that the weekly schedule of carboplatin and paclitaxel was less toxic than and potentially superior to the standard tri-weekly schedule. However, further modification of the dose and schedule is warranted.

## Figures and Tables

**Table I:** Patient characteristics.

Characteristics	Weekly arm (n=73)	Standard arm (n=94)	P-value
Age (years)			0.2361
Median	64	65.5	
Range	31–78	32–79	
Gender			0.3723
Female	21	21	
Male	52	73	
ECOG PS			0.5402
0–2	61	85	
3 or 4	12	9	
Smoking history			0.7300
Never smoked	19	27	
Past or current smoker	54	67	
Histological diagnosis			0.1581^a^
Squamous cell carcinoma	15	29	
Adenocarcinoma	57	61	
Large cell carcinoma	1	3	
Other	0	1	
Initial clinical stage			0.5890
IIIB	19	21	
IV	54	73	
EGFR-TKI treatment			0.2667
Not used	41	60	
Used	32	34	
Objective response			
CR	1	2	
PR	26	28	
SD	27	40	
PD	19	24	
ORR (%)	37.0	31.9	0.5143
DCR (%)	74.0	74.5	1.000

ECOG, Eastern Cooperative Oncology Group; PS, performance status; EGFR-TKI, epidermal growth factor receptor tyrosine kinase inhibitor; CR, complete remission; PR, partial remission; SD, stable disease; PD, progressive disease; ORR, objective response rate; DCR, disease control rate.

a Squamous vs. non-squamous cell carcinoma.

**Table II:** Adverse events (≥grade 2) according to the treatment schedule.

	Weekly arm (n=73)				Standard arm (n=94)				
Toxicity	G2	G3	G4	≥G3 (%)	G2	G3	G4	≥G3 (%)	P-value
Hematological									
Neutropenia	15	19	8	37.0	6	25	41	70.2	<0.0001^a^
Thrombocytopenia	7	5	3	11.0	22	8	4	12.8	0.8127
Anemia	34	9	6	20.5	38	17	9	27.7	0.4651
Nonhematological									
Neuropathy	0	0	0	0.0	9	5	3	8.5	0.0188^a^
Transaminase	7	1	0	1.4	5	4	0	4.3	0.3875
Total bilirubin	0	0	0	0.0	5	2	1	3.2	0.2574
Serum creatinine	3	0	0	0.0	5	0	0	0.0	n.d.

a P<0.05. G, grade; n.d., not done.

**Table III:** Univariate analysis of OS.

Variable	MST (months)	P-value
Weekly arm vs. standard arm	11.8 vs. 11.6	0.3779^b^
Female vs. male	17.1 vs. 10.4	0.0051^a^
Never smoked vs. smoker	16.2 vs. 10.0	0.0043^a^
Age <70 vs. ≥70 years	11.6 vs. 11.8	0.6135^b^
PS 0/1/2 vs. 3/4	12.5 vs. 3.2	0.0062^a^
Non-sq vs. sq	12.6 vs. 9.7	0.0280^a^
TKI used vs. never used	16.5 vs. 9.7	0.0007^a^
Stage IIIB vs. IV	14.6 vs. 10.7	0.0248^a^
Neutropenia (G3 or G4 vs. G1 or G2 vs. G0)	13.3 vs. 10.7 vs. 9.7	0.0243^a^
Anemia (G3 or G4 vs. G1 or G2 vs. G0)	4.7 vs. 12.8 vs. 23.0	0.0002^a^
Thrombocytopenia (G3 or G4 vs. G1 or G2 vs. G0)	5.2 vs. 12.6 vs. 10.6	0.3718^b^

a P<0.05.

b not significant. OS, overall survival; MST, median survival time; PS, performance status; Sq, squamous cell carcinoma; TKI, tyrosine kinase inhibitor; G, grade.

**Table IV:** Multivariate analysis of OS.

Covariate	HR	95% CI	P-value
Weekly arm vs. standard arm	0.634	0.422–0.948	0.0262^a^
Female vs. male	0.528	0.289–0.947	0.0320^a^
Never smoked vs. smoker	0.727	0.415–1.255	0.2557^b^
Age <70 years vs. ≥70 years	1.078	0.714–1.647	0.7245^b^
PS 0/1/2 vs. 3/4	0.296	0.167–0.550	0.0002^a^
Non-sq vs. sq	0.957	0.610–1.526	0.8501^b^
TKI used vs. never used	0.495	0.327–0.744	0.0007^a^
Stage IIIB vs. IV	0.485	0.301–0.756	0.0011^a^
Neutropenia			
(G3 or G4 vs. G0)	0.372	0.215–0.654	0.0007^a^
(G1 or G2 vs. G0)	0.825	0.448–1.527	0.5392^b^
(G3 or G4 vs. G1 or G2)	0.450	0.281–0.728	0.0013^a^
Anemia			
(G3 or G4 vs. G0)	9.527	3.415–29.301	<0.0001^a^
(G1 or G2 vs. G0)	3.514	1.422–9.775	0.0056^a^
(G3 or G4 vs. G1 or G2)	2.711	1.641–4.404	0.0001^a^
Thrombocytopenia			
(G3 or G4 vs. G0)	0.870	0.424–1.709	0.6290^b^
(G1 or G2 vs. G0)	0.785	0.525–1.177	0.2407^b^
(G3 or G4 vs. G1 or G2)	1.108	0.570–2.047	0.7538^b^

a P<0.05.

b not significant. OS, overall survival; HR, hazard ratio; CI, confidence interval; PS, performance status; Sq, squamous cell carcinoma; TKI, tyrosine kinase inhibitor; G, grade, ns, not significant.
